# Positron Emission Tomography in the Inflamed Cerebellum: Addressing Novel Targets among G Protein-Coupled Receptors and Immune Receptors

**DOI:** 10.3390/pharmaceutics12100925

**Published:** 2020-09-28

**Authors:** Margit Pissarek

**Affiliations:** Institute of Neuroscience and Medicine, INM-5, Forschungszentrum Juelich GmbH, D-52425 Juelich, Germany; m.pissarek@fz-juelich.de

**Keywords:** inflammation, positron emission tomography, small molecules, cerebellum, purine receptor, chemokine receptor, immune receptor, peptide receptor

## Abstract

Inflammatory processes preceding clinical manifestation of brain diseases are moving increasingly into the focus of positron emission tomographic (PET) investigations. A key role in inflammation and as a target of PET imaging efforts is attributed to microglia. Cerebellar microglia, with a predominant ameboid and activated subtype, is of special interest also regarding improved and changing knowledge on functional involvement of the cerebellum in mental activities in addition to its regulatory role in motor function. The present contribution considers small molecule ligands as potential PET tools for the visualization of several receptors recognized to be overexpressed in microglia and which can potentially serve as indicators of inflammatory processes in the cerebellum. The sphingosine 1 phosphate receptor 1 (S1P1), neuropeptide Y receptor 2 (NPY2) and purinoceptor Y12 (P2Y12) cannabinoid receptors and the chemokine receptor CX3CR1 as G-protein-coupled receptors and the ionotropic purinoceptor P2X7 provide structures with rather classical binding behavior, while the immune receptor for advanced glycation end products (RAGE) and the triggering receptor expressed on myeloid cells 2 (TREM2) might depend for instance on further accessory proteins. Improvement in differentiation between microglial functional subtypes in comparison to the presently used 18 kDa translocator protein ligands as well as of the knowledge on the role of polymorphisms are special challenges in such developments.

## 1. Introduction

More than half a dozen of consensus papers have been released during the last decade in a wave of stepwise revision of the former restriction of cerebellar contributions to brain function to regulation of movement control [1,2,3,4,5,6].These reviews pay tribute to the multiple functions of the cerebellum in cognitive [1,7], emotional [6] and sensory networks [3]; neuro-immune mechanisms [5]; and linguistic, connectivity, developmental processes [2,4,7,8], and contribute to the internal clock of the body [9] moving the multifunctional cerebellum into the central scope of brain research. Moreover, the view on cytoarchitectonics and regional difference in cellular subtypes of neuronal [10] and of non-neuronal cells [11,12] is attracting increasing attention for this heterogeneity. Purkinje cells are most extensively investigated with cell biological and cell physiological methods, among the seven main types of neuronal cells in the cerebellum [13,14,15]. Microglial cells, key immune cells of the brain and strongly involved in the pruning of neuronal cells and postulated to show altered functional properties in the cerebellum compared to cerebral microglia [16], came late in the focus of brain research and even later in the central scope of positron emission tomographic (PET) neuroimaging methods.

With view on neurodegenerative diseases, the involvement of cerebellar and inflammatory aspects in radiological in vivo imaging approaches opens new kind of morphological access and functional understanding even in widely known hereditary disorders [5,17]. More and more attention is paid to neuro-inflammation in the earlier steps of neurodegenerative but also traumatically and of auto/immunologically induced brain diseases [5,18,19] regarding the limbic system and the cerebellum as preferred targets of autoimmunity.

Diseases with inflammatory features, such as multiple sclerosis, amyotrophic lateral sclerosis, and encephalitis with consequences to movement processing or clinically engraving symptoms such as tremor, are known for a close relation to cerebellar structures. Meanwhile, also rapid alterations of cerebellar microglia in aging processes [20] are a recognized and accepted paradigm and a new challenge to more detailed investigations of pre-inflammatory mechanisms. An accepted feature of cerebellar microglia is the persistence of the immature ameboid phenotype also in the mature brain, which can be found not only in the cerebellar cortex but also in white matter areas [16]. Moreover, the cerebellum is regarded as a model for investigation of brain development, especially of the role of epigenetic factors in this process [21]. This is attributed to the fact that the number of different cell types is relatively small in the cerebellum, and the stages of cerebellar development have been well characterized already for many years [21]. From the topographical point of view, however, emission tomographic in vivo imaging and even magnet resonance imaging remain a special challenge with the close folding of cortical regions in the adult cerebellum [22,23]. Regarding the literature during the last years, microglia quickly became the focus of research, but the number of novel biomarkers characterized also for conditions potentially allowing the in vivo visualization with compounds suitable for emission tomographic methods is very limited.

Baldacara et al. [2] published in 2015 an initial general insight in consensus on radiological markers focused on cerebellar investigations. In this review, investigations of the 5HT [24,25] system as well as [^18^F] fluorodeoxyglucose (FDG) as a metabolic tracer are emphasized. Finally, SPECT and PET imaging play a subordinated role only in this contribution, and inflammatory processes are yet not involved [2].

**Figure 1:**
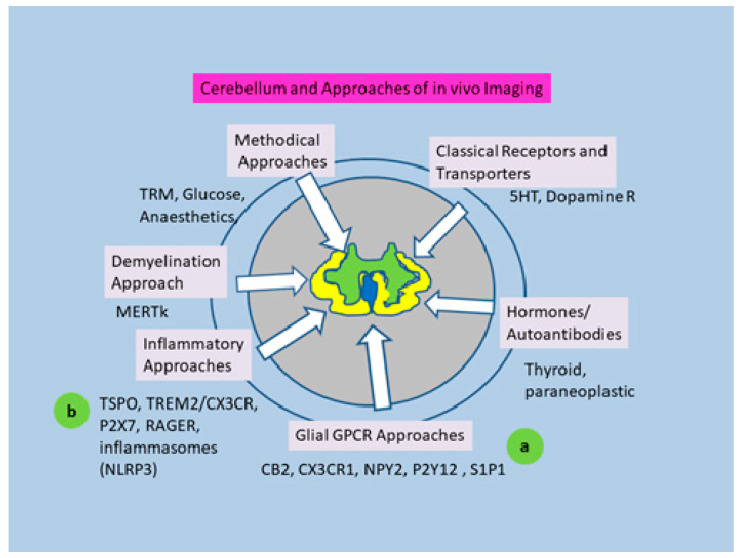
Approaches to cerebellar in vivo imaging reflected by the literature through the last decade. Early approaches concerned metabolic imaging using [^18^F] fluorodeoxyglucose (FDG) or ^15^O, supplemented more recently by respective magnet resonance imaging methods providing increasingly detailed functional and higher anatomic resolution. Classical neuronal receptors visualized also in the cerebellum included, for example, 5 hydroxytryptamine, dopamine, opioid and adenosine receptors. The present contribution is focused on: (**a**) glial G protein-coupled receptor (GPCR) approaches: G-protein-coupled receptors known for inflammation triggered overexpression in microglia and potential small molecule ligands suitable for PET visualization of the receptors; (**b**) inflammatory approaches: the chemokine receptor, the ionotropic receptor and the immune receptors RAGE and TREM2 with influence on the formation of inflammasomes and the current possibilities of visualization with PET. CB2: cannabinoid receptor 2; CX3CR1: chemokine receptor CX3C 1; NPY2: neuropeptide Y receptor 2; MERTK: myeloid-epithelial reproductive tyrosine kinase; 5-HT: 5-hydroxytryptamine; P2Y12: purinoceptor Y12; P2X7: purinoceptor 2X7; S1P1: sphingosine 1 phosphate receptor 1; RAGE: receptor of advanced glycation end products; TREM2: triggering receptor expressed on myeloid cells 2; TSPO: translocator protein 18 kDa.

The recent plurality of reports concerned with in vivo imaging of cerebellar function use MRI methods [17,26] and [^18^F]FDG investigations [27] (see Figure 1). However, more and more details on subregional cerebellar functional patterns are provided.

Narayashwani et al. [28] published in 2018 an overview on targets potentially important as biomarkers of inflammation. The authors included the common translocator protein (TSPO) ligands of several generations and in four structural groups as the type of PET tracer with broadest clinical applications hitherto.

Moreover, the authors highlighted some groups of PET radiotracers in early-stage preclinical and clinical research targeting potential biomarkers of neuro inflammation on an enzymatic and receptor level known to belong to the inflammatory response of microglia, astrocytes and lymphocytes [29].

Several types of G protein-coupled receptors (GPCR) came into the focus as targets of drugs and tracers potentially suitable for in vivo imaging of inflammatory processes and especially of pro- or anti-inflammatory activation of microglia (See Figure 1).

These candidate structures include sphingosine 1 receptors, especially the most abundant subtype, sphingosine 1 phosphate receptor 1 (S1P1); the purine receptor P2Y12; cannabinoid receptors, especially the cannabinoid receptor 2 (CB2) subtype; the chemokine receptor CX3CR1 [30,31]; and the neuropeptide Y receptor 2 (NPY2) receptor. Apart from the ligands of 7TM receptors, regulatory transmembrane, monomeric and homo-dimeric receptor structures are gaining attention of the research community. Notably, the ATP (adenosine triphosphate) gated ion-channel purinoceptor 2X7 (P2X7) triggered the release of compounds potentially suitable as PET tracers and subjected to initial clinical tests [32,33].

Two immune receptor structures, the receptor for advanced glycation end products (RAGE) [34,35] and the triggering receptor expressed on myeloid cells 2 (TREM2) [36,37], prevalent in healthy peripheral and central nervous tissues and changed under pathophysiological conditions, are new kinds of potential in vivo markers also on microglial cells and in different brain areas.

**Figure A1:**
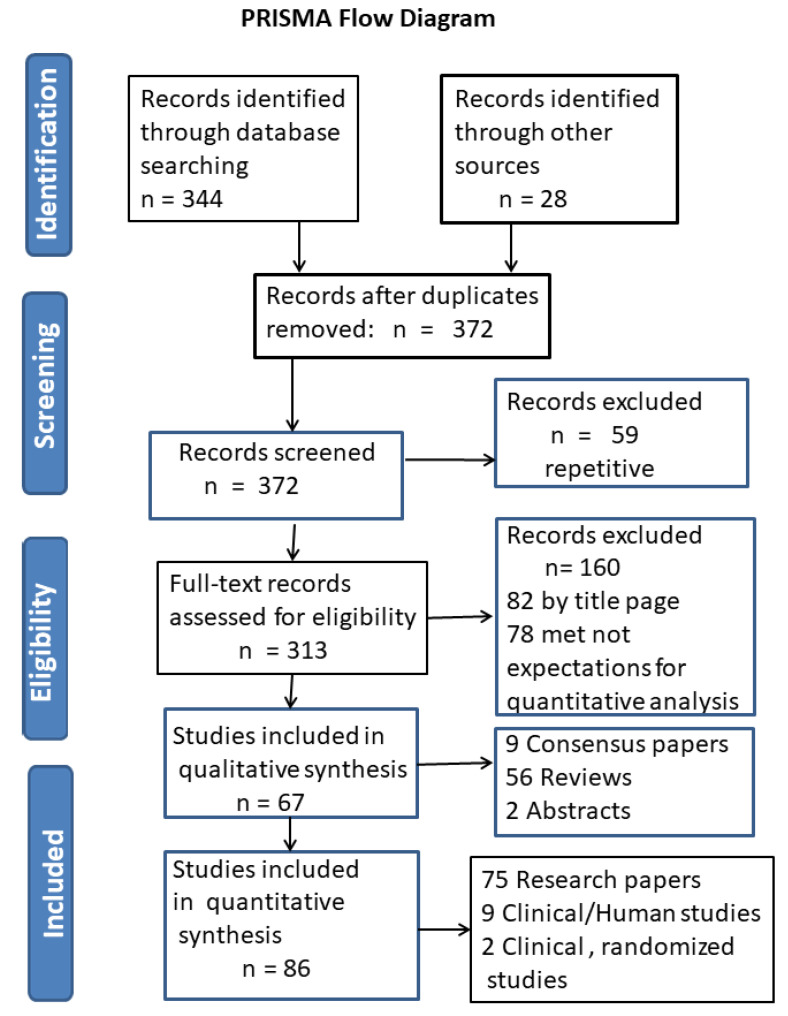
PRISMA flow diagram for studies including qualitative and quantitative analysis. PRISMA, preferred reporting items for systematic reviews and meta-analyses. Modified flow diagram according to [154].

The objective of the current contribution is an update on the efforts for lead structures and small molecule ligands as probes for such potential microglial biomarkers and on their suitability as PET tracers for cerebellar processes (For literature database analysis see also flow diagram in Appendix B, Figure A1).

## 2. Target Receptors and Ligands

### 2.1. Sphingosine 1 Phosphate Receptors

One of the proposed GPCR targets for imaging of inflammation is the S1P receptor. It belongs to the Class A GPCRs [38]. Five subtypes of these receptor interacting with S1P (sphingosine 1 phosphate) as one of the endogenous ligands have been identified to date [39,40]. While the subtypes 1–3 are omnipresent in the body and have been detected in cerebral as well as cerebellar glia cells [41,42], subtypes 4 and 5 are expressed only in few kinds of cells [43], i.e., S1P5 receptor in the brain in oligodendrocytes only [41]. G_i_ protein has been confirmed for signaling with all five subtypes. Additionally, G_q_ is associated with S1P3 and S1P4 subtype, and G_12/13_ with all subtypes except S1P1. Finally, the sphingosine 1 phosphate receptors remain pleiotropic transducers which can influence proliferation, inflammation, anaphylaxis and cancer in both positive and negative directions, frequently dependent on the kind of cell which is the site of the respective subtype.

The S1P1 receptor is among these subtypes that are most abundant in the brain. S1P contains a polar head group and a hydrophobic alkyl chain [44]. Its receptor is regarded as a modulator of cell survival, proliferation and motility in glial cells as well as of epigenetics, aging and mitochondrial respiration [44,45]. An agonistic action of S1P at the S1P1 and S1P3 subtype can induce, here too, different effects. Thus, actions of the S1P1 receptor are related to racGTPase-dependent activation of the cytoskeleton barrier function. The S1P3 receptor supports rohGTPase-regulated cytoskeleton rearrangement and disruption of the barrier function [43].

The most interesting action of S1P1 agonists promising high clinical relevance was the ability to induce immunosuppression within a short time and at low concentrations [46]. Agonists inhibit lymphocyte segregation, the withdrawal of lymphocytes from the circulation and its egress from lymphoid organs to stroma. However, these mechanisms have been reported also to be complex, and agonism can mediate also S1P1 downregulation by functional antagonism.

The S1P2 receptor, which was not a classical target of the first S1P receptor agonists and antagonists, has been described in vascular and immune cells and is involved in the regulation of vascular permeability and the blood–brain barrier function. Moreover, the receptor has been reported to be expressed as sex-and strain-dependent in mice models of multiple sclerosis. It belongs to the few cerebellar loci identified as relevant for autoimmune diseases [47].

Finally, functional effects of different subtypes of the S1P receptors can be synergistic but also antagonistic. S1P is carried from cytosol into the extracellular compartment notably by two transporters, the spinster homolog 2 and an ABC transporter. It acts, subsequently, in autocrine or paracrine mode as an extracellular ligand. S1P-stimulated Ca^2+^ signaling has been described in cultured cerebellar astrocytes as a trigger of neuronal response [41]. The S1P level in blood is 100-fold higher than the commonly used dosage of agonistic drugs at the S1P receptors applied in multiple sclerosis [48].

S1P has been demonstrated to induce apoptosis also in hippocampal and, specifically, in S1P lyase lacking neurons. Cerebellar neurons with originally high expression of S1P lyase initially degenerate in S1P lyase-deficient mice [49]. Intracellular S1P can influence histone deacetylase (HDAC) ½, human telomerase transcriptase (hTERT) and prohibitin 2.

#### 2.1.1. S1P Receptor Ligands with Potential Implication as a PET Tracer

The first small-molecule compounds revealed as ligands of the sphingosine 1 receptor (subtypes 1, 3, 4, and 5) were agonists and derivatives of myriocin (thermozymocidin) a non-proteinogenic, immunosuppressive amino acid obtained from the thermophilic fungus Isaria sinclairii [50,51]. Fingolimod (Gilenya, Novartis) was the first-in-class compound established clinically as an oral prodrug, binding as an active, phosphorylated molecule to all S1P receptor subtypes, except the S1P2 subtype, and it serves as an immunomodulator in the treatment of multiple sclerosis [52]. The biological active compound is the S-enantiomer [50,51].

The change in the core structure of phosphonates and the introduction of a vinyl chain resulting in chirality of the molecule improved the stability and provides possibilities for enhancement of receptor affinity and desired pharmacological effects [43].

Regardless of useful physicochemical properties, the problem of early-response bradycardia [53,54] and general pharmacokinetically triggered efforts for the development of competitive as well as allosteric antagonists. Some of these compounds are also derivatives of sphingosine-1-phosphate [43,55], despite the fact that the pool of antagonists remains limited [43,44].

**Figure 2:**
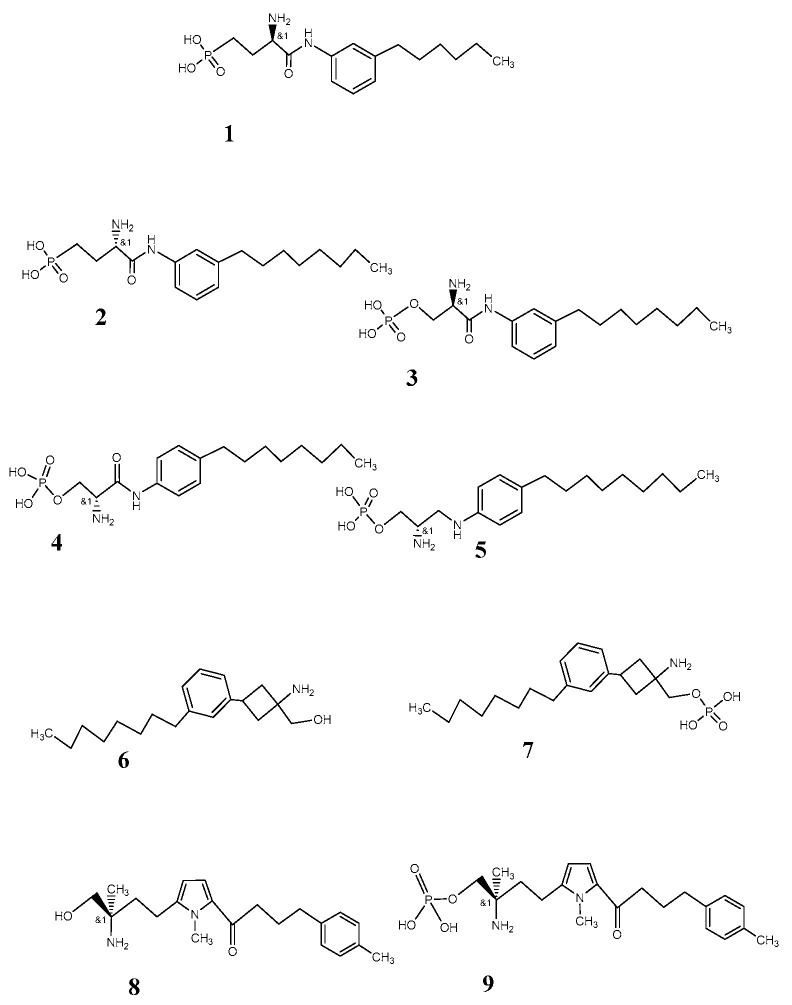
S1P1 ligands **1**: W146 (ML056); **2** VPC4416; **3** VPC 23019; **4** VPC 22173; **5** VPC22277; **6** VPC 3090; **7** VPC 3090P; **8** CS-0777; **9** CS-0777P. (For IUPAC names of all compounds according to ChemDraw 19.0 see Appendix A)

**Figure 3:**
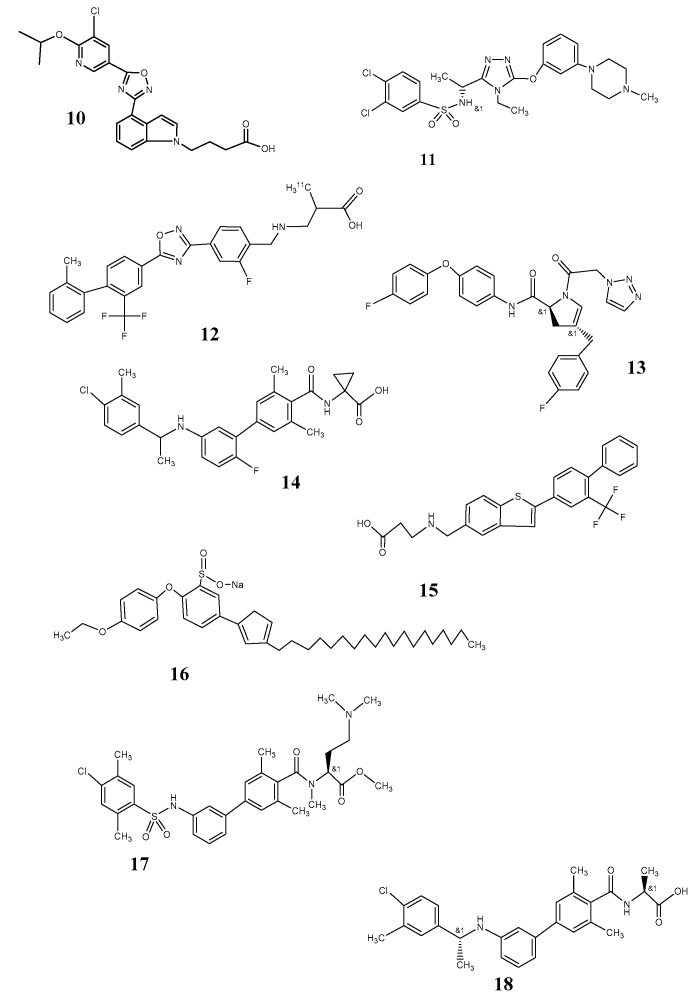
S1P receptor ligands **10**: GSK2018682; **11** TSAP0277308; **12** TZ3321; **13** XLS541; **14** Ex26; **15** AUY954; **16** CL2; **17** Prodrug 14; **18** NIBR0213.

The first antagonist at the S1P1 receptor subtype, W146 (ML056; Figure 2, [1]) [41,51,52,53], and the antagonist VPC44116 [2] showed high selectivity for the subtype. These antagonists, as well as the compound VPC23019 (Figure 2, [3]), have the moderate lipophilic properties necessary for a drug or diagnostic compound for parenteral application in imaging of brain function. Two candidates among the fingolimod derivatives of second generation, VPC23019 and VPC03090, respectively their phosphorylated derivatives, show additional specificity for subtypes 3 of the receptor (Figure 2 [6,7]).

The pyrrole compound CS-0777 (Figure 2: [8,9]) provides also log P in an appropriate range for the blood–brain barrier passage (2.63). Although it is an agonist at the S1P1 and S1P3 subtypes of the sphingosine 1 phosphate receptor [54,55,56,57], the selectivity at S1P1 vs. S1P3 is approximately 10-fold that of fingolimod. Half maximal effective concentrations (EC_50_) for agonist-induced binding of [^35^S]GTPγ-S to human S1P1 and S1P3 receptors overexpressed in chinese hamster ovary (CHO) cells wereat 1.1 and 1.8 nM (S1P1) as well as at 200 nM and 350 nM (S1P3) in comparison to fingolimod (EC_50_: 0.29 and 0.37 nM, S1P1; EC_50_: 1.3 and 3.3 nM, S1P3) [57]. Moreover, CS-0777 confirmed in an experimental autoimmune encephalitis (EAE) Lewis rat model potential efficacy in treatment of multiple sclerosis. W146 and VPC23019 provide similar properties with respect to log P.

All the related derivatives deduced from this structure act as prodrugs activated by sphingosine kinases in vivo [53]. Most of them induce a reduction in circulating lymphocytes and an early-response bradycardia [48,53,54]. Regarding the subtype selectivity of the pharmaceutical, today, there are further compounds available with higher binding affinity and, notably, also with higher selectivity for the abundant subtype S1P1 [48,51]. Fingolimod has been now defined as a receptor agonist, but a functional antagonist, which is characteristic of a modulator rather than of a specific interaction at the S1P1 receptor [48].

Among the next-generation small molecule compounds with specific affinity to S1P1, Ozanimod (RPC1063, Celgene) and GSK 2018682 (GlaxoSmithKline) (Figure 3, [10]) are prominent because of their high subtype selectivity in comparison to fingolimod (10,000 fold for S1P1 vs S1P3). Ozanimod is able to cross the bbb (blood–brain barrier) and achieves EC_50_ between 0.16 and 0.41 nM depending on the methodical approach. It has a markedly shorter biological half-life than fingolimod [43] and promises also reduced side effects. Moreover, Ozanimod and GSK2018682 show appropriate log D (2.32 and 2.61) for actions also in brain tissue. Both are, however, regarded as modulators of the receptor rather than as competitive ligands [53].

Some of the S1P1 antagonists of the third generation, structurally deviating from sphingosine 1 phosphate as a lead structure, have been demonstrated convincing therapeutic effects on inflammatory processes in animal models. This was reported for TASP-0277308 (Figure 3: [11]) with a reduction in extent of arthritis in mouse models, especially mediated by a decrease in T-cell infiltration [48]. However, molecular weight as well as log P are in a rather broader range of pharmacokinetic requirements. In general, modulators with higher selectivity for S1P1R are regarded to trigger via G_i_ proteins, Gproteincoupled inwardly rectifying potassium channel (GIRK)-mediated bradycardia, and endothelial NO synthase (eNOS)-mediated decline in blood pressure, but prevent also plasma leakage from blood vessels and protect the endothelial barrier function mediated via the pathway of proteinkinase B (Akt) and rac GTPase involved in regulation of the actin cytoskeleton and of cellular movement [48]. In contrary, S1P2 and S1P3 selectivity is paralleled by vasoconstriction and impairment of the endothelial barrier function mediated via rho/rock non-G_i_ signaling [48].

While there is a multitude of further agonists of herbal origin, small molecules which can act as antagonists are rather limited. PET in vivo investigations have been performed with focus on vascular inflammation using ^11^C-labeled TZ3321 (Figure 3: [12]). However, the oxadiazol is generally too lipophilic (log P 7.24) for a use as a tracer for specific brain imaging [58].

Several antagonists have been tested in experimental models like EAE, and some achieved also clinical test phases, especially for therapeutic indications in multiple sclerosis [44]. While the S1P1 antagonist EX26 (Figure 3: [14]) could not enter into brain tissue, NIBR0213 (Figure 3: [18]) was able to cross the blood–brain barrier [48].

#### 2.1.2. S1P2 Ligands

The affinity of S1P is a few nmoles lower at the S1P2 receptor than at the S1P1 receptor [59]. Functionally, the S1P2 receptor also coordinates a one-directional signal mechanism that is not simple. Activation of S1P2R can result in pro-inflammatory but also in anti-inflammatory consequences. Moreover, pro-and anti-cancerous actions have been described. The S1P2 has been suggested as a receptor for binding lipids as well as proteins for activation [60].

**Figure 4:**
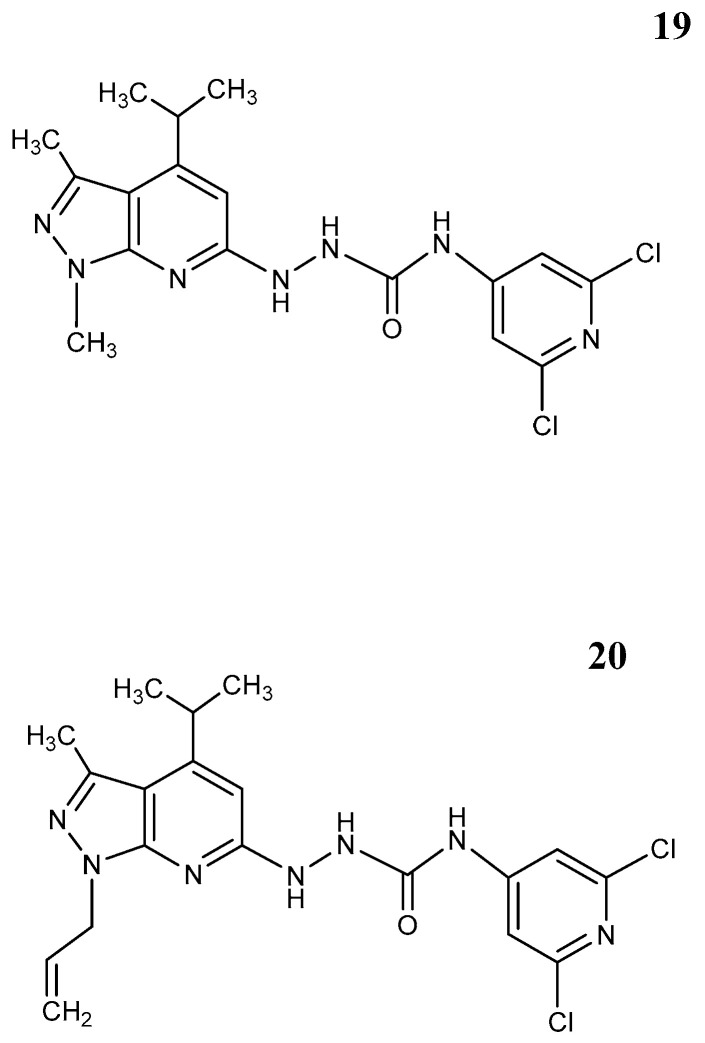
S1P2 receptor antagonists. **19** JTE 013; **20** AB1.

The S1P2 antagonist JTE 013 (Figure 4 [19]) has been shown to influence angiogenic sprouting, smooth muscle of airways as well as integrity of blood vessel barriers through Gα_12/13_ [59,61]. JTE-013, however, shows low stability in vivo. This was improved with its derivative AB1 [61] (Figure 4: [20]). The main application for these compounds was recently seen in treatment of neuroblastoma at the level of experimental models.

A series of 1.3 bis-(aryloxy) benzene derivatives was provided by Kusumi et al. [62]. A clinically useful S1P2 antagonist, is, however, not available to date, although such an option is regarded as a valuable supplement of tools for diagnostics and research.

### 2.2. Cannabinoid Receptors

Cannabinoid receptors are also class A G-protein-coupled (7TM) receptors which mediate signaling, primarily via G_i/0_-proteins or G_q_ proteins, and are activated by endocannabinoids. Further endogenous ligands of the CB receptors are anandamide, sphingosine 1 phosphate, 2-arachidonyl glycerol and sphingosylphosphorylcholine [63]. While the CB1 subtype is regarded as responsible for psychoactive actions of endocannabinoids, the CB2 receptor has been shown to be involved in inflammatory processes [64] and is expressed in macrophages and microglia. For agonist actions related to treatment of pain symptoms, a lower development of tolerance for CB2 specific drugs is of special interest and, potentially, of advantage in clinical application [64]. In general, the effects triggered via cannabinoid receptors in different organs are numerous [65]. The CB1 subtype is expressed predominantly presynaptically and in a high density [66] compared to the CB2 subtype, which is expressed postsynaptically [67]. Presence of cannabinoid receptors also in the cerebellum is well-know and has been investigated early in the monkey brain and also in the human brain [68]. Cannabinoid receptors have an extremely wide spectrum of relations to diverse diseases of brain and peripheral organs [65].

The hippocampus, cerebellum, basal ganglia and olfactory cortex are typical brain locations of the CB1 subtype. CB2 mRNA has been found using in situ hybridization in brains of non-human primates in the globus pallidum and using RT-PCR analysis in the cortex, hippocampus, striatum, cerebellum and retina [67]. Even if the density of CB1 receptors in the brain is higher than for CB2 receptors, CB2 receptors are induced under particular conditions such as inflammation. Further non-CB1 and non-CB2 targets of cannabinoid are discussed in the literature [69].

#### Cannabinoid Receptor Ligands

**Figure 5:**
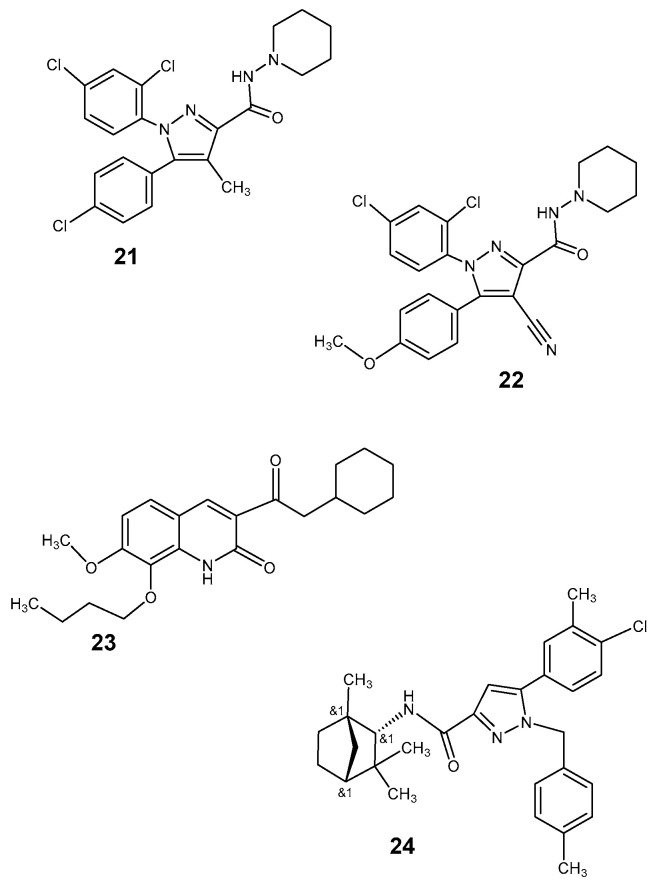
Cannabinoid receptor ligands. CB1: **21** SR141716 (Rimonabant); **22** [^11^C] JHU-75528; CB2: **23** NE40; **24** SR144528 (CB2).

The first cannabinoid receptor antagonist introduced in clinical use was rimonabant (SR141716A) (Figure 5: [21]) [70] shown, finally, also as an inverse agonist. From a therapeutic point of view, it has been described effective in several clinical indications such as weight reduction in obesity [63] and improvement of the lipid profile in obesity and diabetes, as well as in tobacco weaning. The rimonabant derivative [^11^C] JHU75528 (Figure 5: [22]) was an initial PET ligand for the CB1 receptor [71]. The fluorine-18 labeled compound MK9470, a CB1 receptor ligand with 60-fold higher affinity to CB1 than to CB2 receptor, was investigated by Burns et al. [68] with PET. The uptake observed in two monkeys was high in the investigated brain regions including also the cerebellum, with ratios of specific to nonspecific binding of 4–5:1 (displacement with MK0364) and half maximal inhibitory concentrations (IC_50_) at CB1 receptors of 0.7 nM and of 44 nM at CB2 receptors. Humans showed intermediate uptake in the cerebellum and lowest values in the thalamus and hippocampus [68].

Alkylamides (from Echinacea angustofolia or *Othantus maritimus* L.) with structural similarity to anandamide have higher affinities to the CB2 receptor than endocannabinoids and show higher selectivity for the CB2 subtype when compared with CB1 receptor, respectively. Amyrin, as a steroid derivative that moves with its anti-nociceptive and anti-inflammatory actions via cannabinoid receptors, is also in the focus of cannabinoid receptor ligand development [65].

While most in vivo measurements in primates focused on the CB1 receptor, the CB2 receptor as an important structure in inducible processes expressed in microglia also stood in the focus of drug and tracer development. While the normal density of this receptor in the brain is lower than for the CB1 subtype, the CB2 subtype has been described as highly inducible in conditions like addiction, inflammation, anxiety or pain [67]. A limiting factor as a therapeutic target is its peripheral expression, which is much higher than in the brain [67]. Ni et al. [72] summarized compounds proposed as ligands of CB2 receptors, including oxoquinolines, thiazoles, oxadion and thiophenes [72].

Evens et al. tested 2011 [73,74] two high-affinity CB2 receptor agonists (with some species differences) also using micro PET investigations. The indole derivative [^18^F] FE-GW405833 was described with higher selectivity in relation to the CB1 receptor subtype and with somewhat more appropriate log D than the oxoquinoline NE40 (Figure 5: [23]). However, the authors gave more support for NE40 as a potential clinically relevant structure due to the presence of some metabolites of FE-GW405833 as potential disturbing factors in PET investigations. The binding behavior related to the CB2 receptor was described in the spleen with a B_max_ of 0.7 pmol/mg protein for rats (standard uptake value (SUV) up to 4) and after injecting the vector of hCB2 receptors in the rat striatum. The brain uptake in rhesus monkey could be demonstrated with microPET for both tracers. Cerebellar uptake was at a similar level as in the prefrontal area.

The first selective antagonist of the CB2 receptor, SR 144258 (Figure 5: [24]), has been described already 1998 by Rinaldi-Carmona et al. [75]. However, this was an orally active antagonist. Derivatives suitable as PET tools have not been published to date.

### 2.3. P2Y12 Receptor

The P2Y12 receptor is also G protein-coupled and mediates intracellular signaling via G_i_ proteins. Adenosine diphosphateis its endogenous agonist. P2Y12 receptor activation results in hyperpolarization which is triggered via phospholipase C and Inositoltriphosphate (IP_3)_-induced Ca^2+^ mobilization from intracellular stores [32,76]. Several small molecule antagonists have been identified and investigated over the years, especially in cellular and electrophysiological models [77]. Clinical applications of P2Y12 inhibitors have been published in the cardiovascular field, especially as inhibitors of thrombocyte aggregation. However, recently, its importance in inflammatory processes is also finding rising attention in regard to bronchial asthma and other lung diseases, atherosclerosis, cancer or in relation to sepsis [78]. Expression of the receptor has been described to be enhanced by 10-fold, and the mRNA increased by about 10- to 100-fold [79].

The presence of the receptor in microglia has been confirmed on the mRNA level and for the protein [80]. The receptor is expressed in the microglial membrane among other purine receptors and adenosine receptors, which also induced anti-inflammatory actions [78].

#### P2Y12 Receptor Ligands

**Figure 6:**
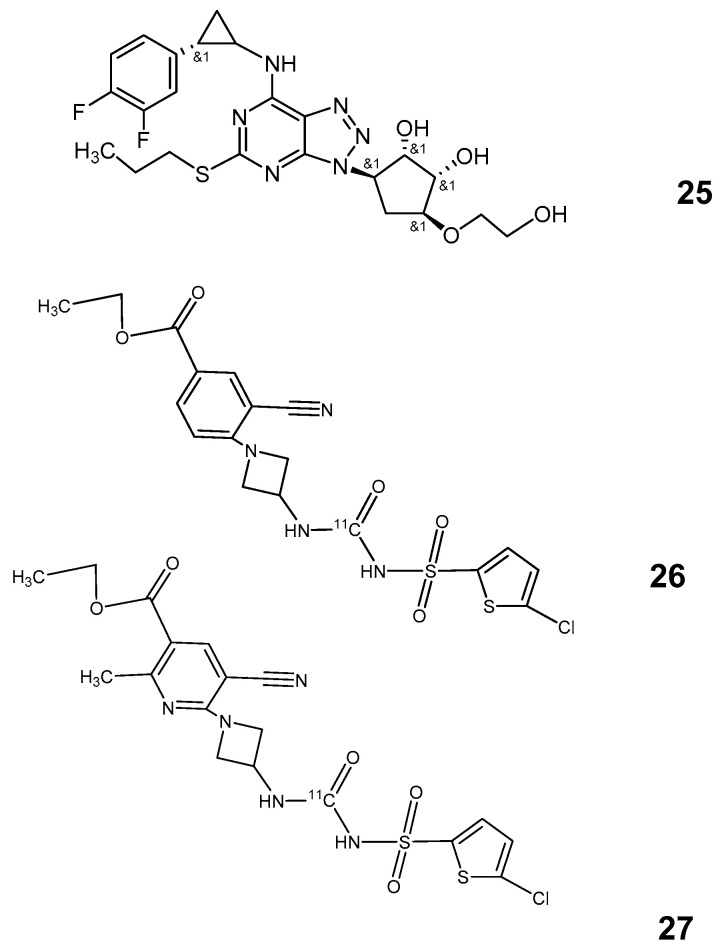
P2Y12 antagonists: **25**: AZD6140; **26**: C2; **27**: C5.

Most of the P2Y12 inhibitors are drugs that are applied orally, such as AZD6140 (Ticagrelor, Astra Zeneca), and established clinically (Figure 6: [25]). Few compounds have been tested also in PET investigations to date. Only one of the clinically used inhibitors (Cangrelor) is suitable for intravenous application [81]. The first P2Y12 antagonist for potential PET investigations was [^11^C] C2 [27] (Figure 6: [26]). Results of the evaluation of an analogue (C5) of this compound were recently published (Figure 6: [27]) [79]. P2Y12 receptors have been shown to be overexpressed in activated M2 type of microglia [28] but reduced in microglia close to plaques of Alzheimer’s disease or lesions of multiple sclerosis. This supports the hypothesis that P2Y12 receptor ligands can differentiate better between the M1 and M2 subtype of microglia and ligands of the 18 kDa translocator protein (TSPO), currently, widely used for identification of microglia with PET methods. Both [^11^C] C2 and [^11^C] C5 show with 2.15 and 2.2 appropriate log P to combine the ability to cross the blood–brain barrier with sufficient selectivity for the receptor. C2, however, showed an extremely low uptake in vivo which was the reason for its exclusion in the further development as a PET tracer [29]. For C5, it was shown that binding was increased in IL4-stimulated tissue but not following stimulation with lipopolysaccharides (LPS). The first oral clinical inhibitor of P2Y12 [82], AZD6140 (Figure 6: [24]), remains in its main indication as an inhibitor of platelet aggregation in contest with clopidogrel, while C5 (Figure 6: [27]) could provide a useful lead structure for further evaluation and development as a tracer of microglial inflammatory activity.

### 2.4. Fractalkine Receptor (CX3CR1)

CX3CR1 is the unique G-protein-coupled receptor among chemokine receptors [83,84,85,86]. The signaling is mediated by the G_q_ protein and PI_3_ kinase pathway. The receptor, generally, is expressed in myeloid cells (macrophages, microglia) [83,84,85,86,87]. The endogenous activator fractalkine has been observed in neurons and endothelial cells. In the central nervous system CNS, the CX3CR1 receptor is expressed predominantly on microglia. Recent reports describe also the role of CX3CR1 in the seeding of circulating cancer cells and suggest an essential impact of receptor antagonists in the treatment of metastatic cancer in clinical course [88]. Among the well-known diseases involving several cerebellar regions, especially the posterolateral neocerebellar cortex and the adjacent archicerebellar cortex, are autism spectrum disorders (ASD) [8], which are related to a decrease in CX3CR1 as well as to some rare genetic variants of this receptor. Moreover, recently, CX3CR1-deficient microglia is presumed to exhibit a premature aging transcriptome and a reduced inflammatory response [89].

Recently, CX3CR1 polymorphism in humans has been observed in 25–30% of the population [30]. Consequently, a decreased adhesion of fractalkine can result in dysregulation of microglial activation [30]. In experimental autoimmune encephalitis, CX3CR deficiency is related to demyelination and an exacerbated mode of the disease [30].

Autoimmune diseases with a demyelination pattern, such as multiple sclerosis or amyotrophic lateral sclerosis and inflammatory diseases like encephalitis, further neuropsychiatric disorders like Tourette syndrome [17], with motoric and vocal tics belonging to the putative conditions with cerebellar involvement and cerebellar alterations of this biomarker of inflammation.

#### CX3CR1 Ligands

The number of endogenous ligands of the receptor is very limited and can be essentially reduced to fractalkine, a protein acting as a transmembrane and also as a fractionated soluble activator of the receptor [90,91].

**Figure 7:**
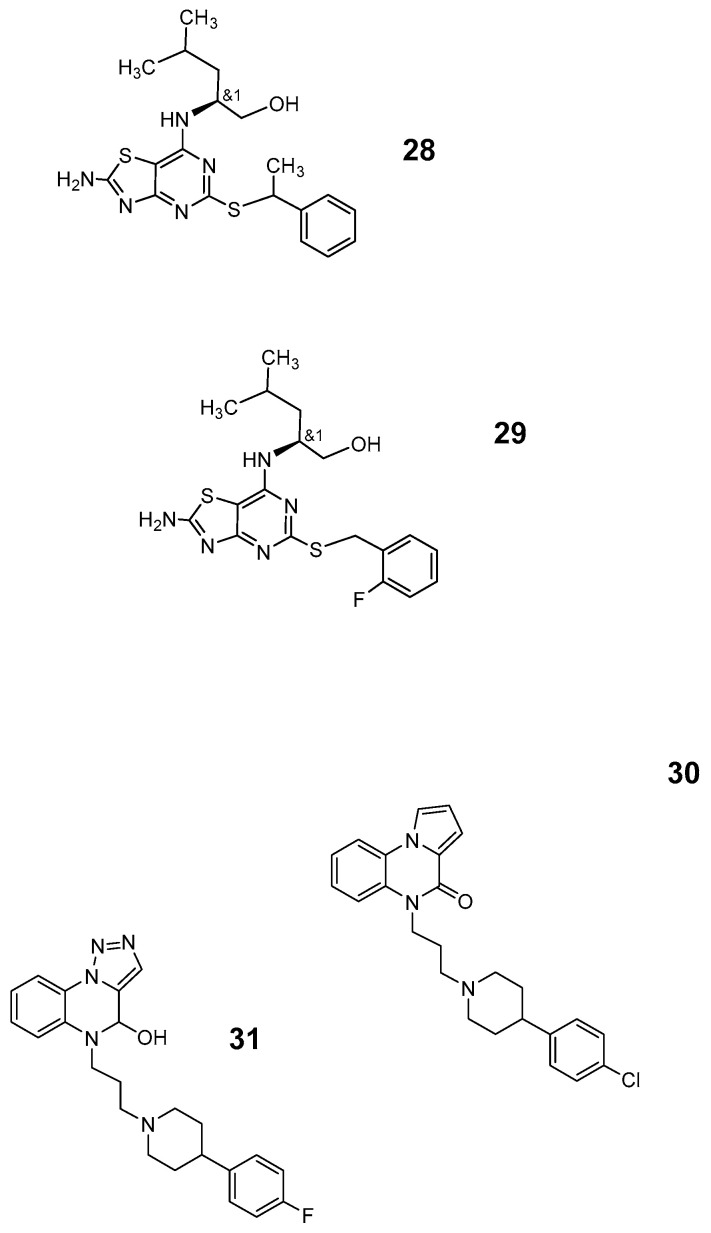
Antagonists of CX3CR1 and allosteric modulators: **28**: AZD8797; **29**: FBTTP; **30**: JMS-17-2; **31**: modified JMS-17-2. (For IUPAC names of all compounds according to ChemDraw19.0 see Appendix A.)

Small molecules have been in series of thio thiazolo pyrimidines (Astra Zeneca) as orally applicable lead structures [85,86,92] such as the allosteric, non-competitive CX3CR1 ligand AZD8797(Figure 7: [28]) developed for treatment of multiple sclerosis. The first F-18 labeled compound for PET applications was released 2015 with the fluorobenzylthiothiazolopyrimidine FBTTP as a derivative of AZD8797 (Figure 7: [29]) [92,93,94].

Finally, also small molecule structures recently proposed as CX3CR1 antagonists are relatively lipophilic. This is true also for JMS-17-2 (Figure 7: [30]) which became known notably for its ability to reduce metastatic seeding in a mouse model with cardiac inoculation of breast cancer cells [88].

The change to appropriate log P can be achieved with few alterations in the tricyclic part of the molecule (Figure 7: [31]) (clogP: 2.5). However, selectivity, affinity and final suitability for brain PET remains to be elucidated.

Notably, marked extravasation of inflammatory cells can be induced in the cerebellum of EAE mice [90]. Blocking the CX3CR1 with a neutralizing antibody or transcriptional construct with CRISPi assistance results in reduced homing of cancer cells to bone and also of dendritic cells detected in bone [88]. Whether JMS-17-2 possibly provides a parent structure also for an effective PET tracer remains to be elucidated.

### 2.5. NPY2 Receptor

The NPY2 subtype of the NPY receptor was, to date, most successfully targeted with PET methods [95] in experimental models. The endogenous 36 a.a.r. neuropeptide Y is the most abundant neuropeptide in the brain. All of the five subtypes of NPY receptors characterized as human receptors are Class A G-protein-coupled receptors and mediate their actions, predominantly, with G_i/o_ proteins [96,97]. NPY2 expression has been described especially during brain development, in neurogenic niches [98,99], and in retinal microglia [100]. Even if initial reports on the presence of NPY receptors in the cerebellum showed rather low values at 3% of total brain labeling [101], initial investigations with an NPY2 receptor ligand with PET demonstrated in animal studies cerebellar uptake comparable with that in the frontal cortex [102].

#### NPY2 Receptor Ligands

**Figure 8:**
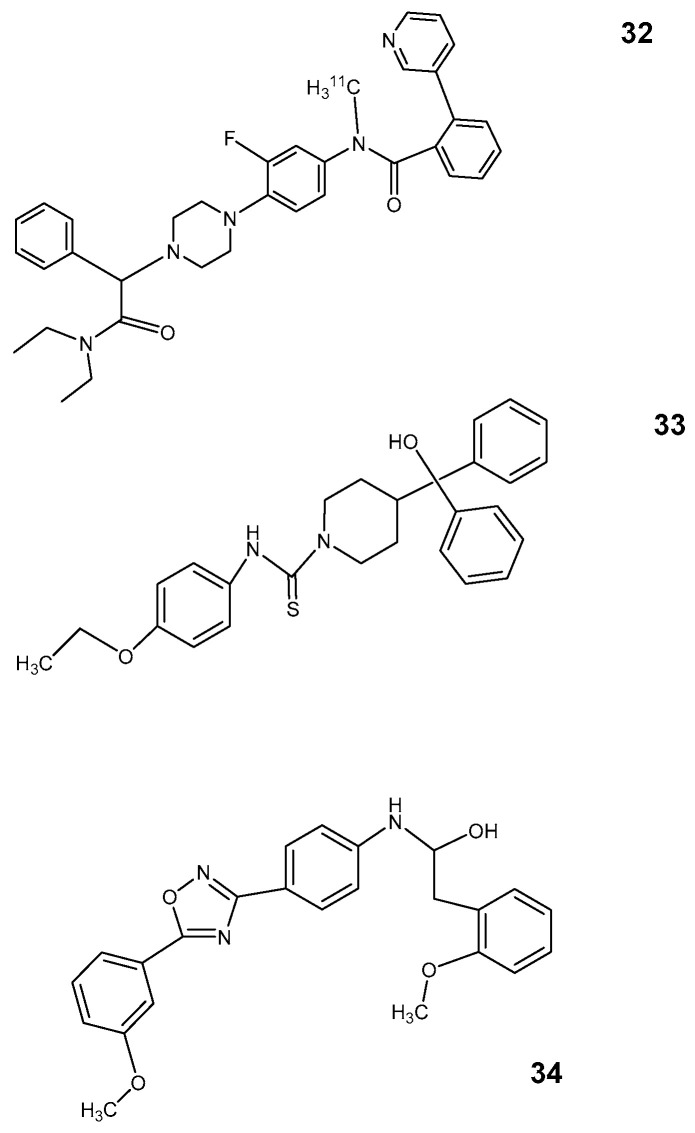
NPY2 ligands: **32**: *N*-^11^C-Methyl-JNJ-31020028; **33**: SF 11; **34**: SF 31.

In 2014, Winterdahl et al. [102] published PET investigation of York Shire land race pigs using the fluorophenylpiperazine derivative *N*-^11^C-Methyl-JNJ-31020028 (Figure 8: [32]). Autoradiographic investigations of the brain in vivo revealed a tracer accumulation in the cerebellum of the healthy pig when compared with that in the basal ganglia or frontal cortex. Higher accumulation was seen in the hippocampus and diencephalon. Two metabolites with faster elution than the parent compound were presumed to be of higher polarity.

The binding of compound 32 in the hippocampus was enhanced three-fold after pretreatment with cyclosporine. This reflected the role of the P-gp transporter in efflux and accumulation of the tracer [102]. The authors described a similar effect of cyclosporine on the tracer accumulation in corpus callosum. However, the challenge with the cold, unlabeled compound revealed specific binding in this experiment only for the gray matter region (hippocampus) and not for white matter (corpus callosum). The authors proposed for that reason corpus callosum as an appropriate reference region in such investigations.

SF 11 (Figure 8: [33]) was one of five compounds presented 2010 by Brothers et al. [103] as potentially brain-penetrating high-affinity NPY2 antagonists. SF 11 is a piperidincarbothiamide, while the other structures were arylsulfamoylbenzoid, aryl-1,2,4-oxadiazol (like SF 31 (Figure 8 [34]) or arylsulfonylmethylisoxazol. SF 11 evaluated further by Domin et al. [104] showed a good uptake into the rat brain. The authors suggest, based on results of forced swimming tests in rats, an anti-depressant-like action of the compound. PET investigations were not part of the study. SF11 and SF31 have a log P at 4.8, which might indicate a reduced selectivity of such compounds in regard to the NPY2 subtype. Test of SF 11 and SF31 for their affinity to classical GPCR receptors [104] showed for SF 31 relatively few interferences but for SF11 also interactions with 5HT receptors or dopamine transporters [104].

### 2.6. RAGE: Receptor for Advanced Glycation End Products

The immune receptor is a 35–45 kDa protein [34,105] with 22 isoforms in humans. It exists in a full-length version and as a soluble version. The full-length version comprises double-sheet extracellular domains, a transmembrane helical domain and a cytoplasmic tail [105].

RAGE is known for pro-inflammatory effects, involvement in the development of vascular leakage, in uptake of Aß amyloid across the blood–brain barrier, Alzheimer’s disease-associated oxidative stress, and in neuronal death [106]. Finally, upregulation of RAGE has been reported to influence cardiovascular diseases, neurodegenerative disease and to promote tumor diseases via the microenvironment and angiogenesis [105].

RAGE can bind intracellular and extracellular endogenous ligands of very different size until they become Aß protein structures. With this ability, it has similarities to pattern recognition receptors also involved in immune response. Small endogenous molecules binding to RAGE are *N*-Carboxy-methyl-lysine (CML) and *N*-carboxy-ethyl-lysine (CEL).

#### Small Molecule RAGE Ligands

**Figure 9:**
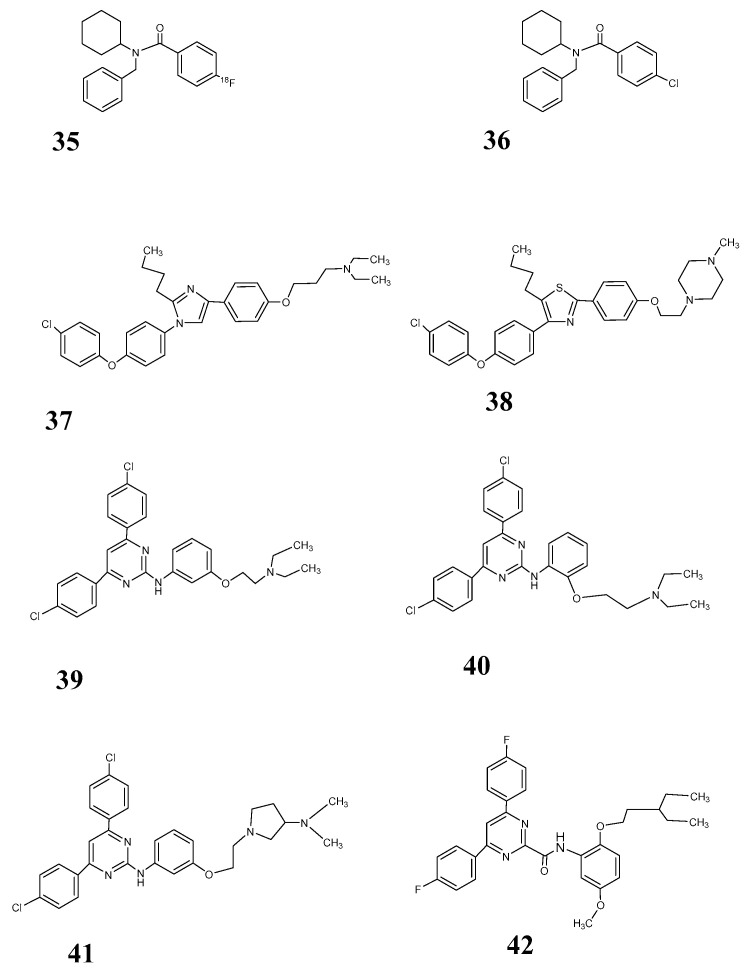
RAGE ligands **35**: [^18^F] RAGER **36**: FPS-ZM1; **37**: azeliragon (TTP488); **38**: cmpd 2; **39**: cmpd 3; **40**: cmpd 4; **41**: cmpd. 5; **42**: cmpd 6.

Initially, the PET ligand for RAGE (receptor for advanced glycation end products) was the fluorobenzamide [^18^F] RAGER (Figure 9: [35]) [34,35]. It is a derivative of FPS-ZM1(Figure 9: [36]) described by Hong et al. [107] as an inhibitor of Aß amyloid metabolism and of inflammation induced by advanced glycation end products (AGE). Its distribution in the brain of non-human primates was investigated with PET, and high accumulation was shown in the cortex as well as the cerebellum, but the highest uptake was observed in the hippocampus [34].

Azeliragon is an antagonist of RAGE already shown to be therapeutically effective in a clinical phase II study. It has been reported to improve cognitive performance, reflected by a reduction of 2.7 points [34,106] on the Alzheimer’s Disease Assessment Scale-cognitive subscale (ADAS-cog11) [108] at the end of a multicenter, randomized, double-blind, parallel study with a low-dose application of azeliragon for 18 month.

All structures proposed as azeliragon derivatives by Bongarzone et al. [105] (Figure 9: [37,38,39,41,42]) share a pharmacophore concept with a heteroaromatic core binding hydrophobic domains and an alkyl linker to a moiety with a protonable nitrogen. However, all these compounds show a logP much too high for their use as a PET tracer. Even as an oral drug, their specificity at the target structure could be in doubt. The properties for this condition are for [^18^F] RAGER and FPS-ZM1 with log P between 4 and 5 also not optimal but somewhat better than for the azeliragon derivatives. For RAGE ligands, at the moment, the pharmacokinetics remains the main challenge for the development of probes for PET imaging.

### 2.7. P2X7 Receptor

The P2X7 receptor belongs to a family of ionotropic receptors [2,109,110,111] and is proposed to play a role in ATP-driven danger transduction [112]. It comprises two transmembrane domains and enables membrane transfer also of larger molecules of up to 800 Da [32,33,76]. ATP stimulates via the P2X7 receptor in microglial cells the formation of pro-inflammatory cytokines like TNFα and IL1β and is presumed to trigger also apoptotic cell death.

The P2X7 receptor is one of the abundant receptors observed in peripheral organs and in the brain [113]. In the periphery are monocytes and macrophages, predominant loci of the receptor. In the brain, microglia is accepted as the predominant site of P2X7 expression [113,114,115]. It has been identified also in the cerebellum even in healthy volunteers. Quantitative autoradiographic measurements in rat brain resulted in B_max_ of 112 fmol/mg × protein, determined for the [^3^H] A804598 [116]. Cerebellar tissue showed approximately two-thirds of the hippocampal binding efficacy. This should be under normal conditions rather than at the threshold of detectability for PET visualization. Quantitative data under conditions of overexpression in wild-type native brain tissue are yet not available.

However, there has been presumed also the presence of a neuronal presynaptic P2X7 receptor based on investigations of receptor mRNA [117]. This is a postulate under debate, but there is absence of evidence for a significant receptor protein expression in neurons [118,119]. Experimental tests of this hypothesis with the aim of identification of the P2X7 receptor on the protein level could not confirm the idea and concluded that P2X7 in neurons is not present in a detectable amount [119].

The P2X7 receptor has been shown in the cerebellum in microglia, astrocytes and Bergmann glia cells [120]. Activation of P2X7 receptors can provide in microglial cells a key signal in the transformation of the inactive NLRP3 inflammasome (nucleotide binding oligomerization domain-containing protein (2NOD), myeloid-epithelial reproductive tyrosine kinase (LRR) and pyrine domain containing protein 3) into its active version by increasing the potassium outward flux, resulting in a decrease in cytosolic potassium [120,121,122]. In this way, P2X7 could amplify pro-inflammatory processes. This is part of the inflammatory response followed by immunosuppression also during sepsis. In mouse models of sepsis with enhanced P2X7 receptor activity increased mortality associated with mitochondrial damage and inhibition of NLRP3 signaling has been shown, suggesting a disturbance of this pathway in such conditions [123]. Such knowledge and also the questions on receptor expression under different pathological conditions triggered intensive efforts also for the development of PET tracers. A multitude of several candidates has been disclosed, and some of these were also included in clinical studies.

#### P2X7 Receptor Ligands

**Figure 10:**
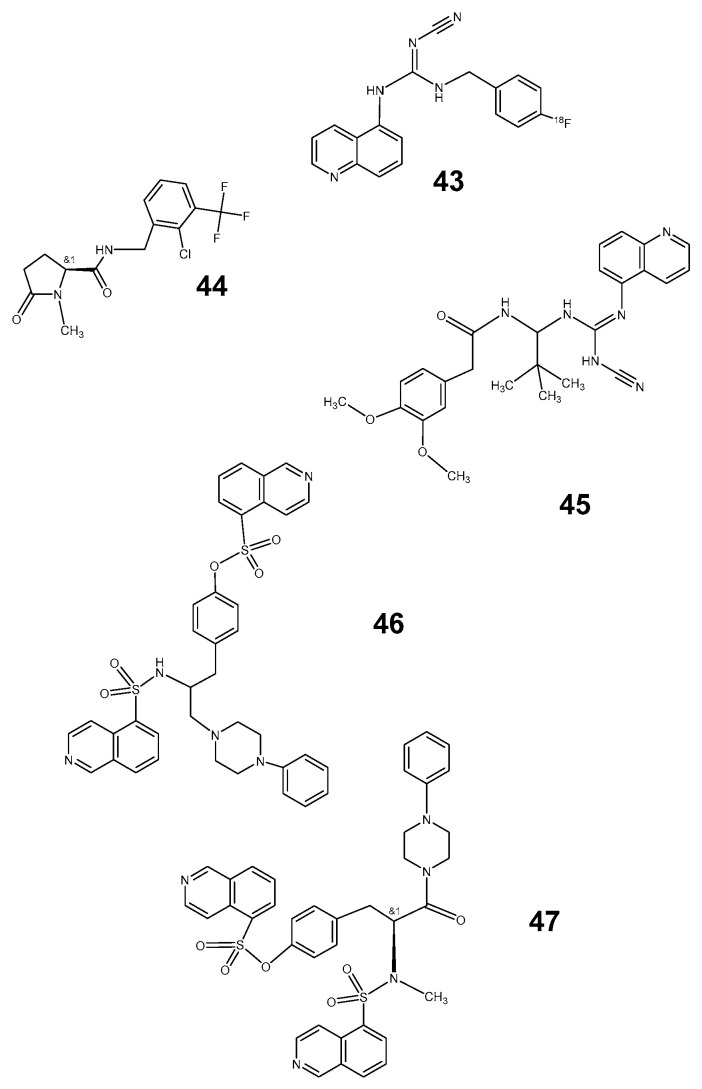
P2X7 receptor ligands **43**: [^18^F] EFB; **44**: [^11^C] GSK1482160; **45**: A-740003; **46**: KN04; **47**: KN62.

Efforts in the development of P2X7 receptor ligands and the labeling with positron emitting isotopes have revealed to date numerous high-affinity compounds [113]. The first radioligand reported for in vivo application was [^11^C] A-74003. (Figure 10 [45]. [^18^F] FEFB (Figure 10: [43]) was the first F-18 labeled PET tracer for this target. Although the spectrum of P2X7 receptor ligands is broader than, for instance, for CX3CR1 and some of them were introduced in clinical trial phases, several of these were discontinued. [^11^C] GSK1482160 (Figure 10: [44]) [124] was recently reported to be accumulated up to 3.2-fold in whole brain PET measurements in lipopolysaccharide-stimulated mice and was selected for evaluation as a PET tracer. However, the following phase 1 safety study was discontinued. [^11^C] A-740003 (Figure 10: [45]) [125,126] and has been recently used by Beiao et al. [127] as a tritium-labeled compound in autoradiography studies of acute and chronic alterations of microglia in a model of multiple sclerosis associated with an antibody study of P2Y12 receptors. KN04 and KN62 are non-competitive antagonists of P2X7 receptors (Figure 10: [46,47]).

**Figure 11:**
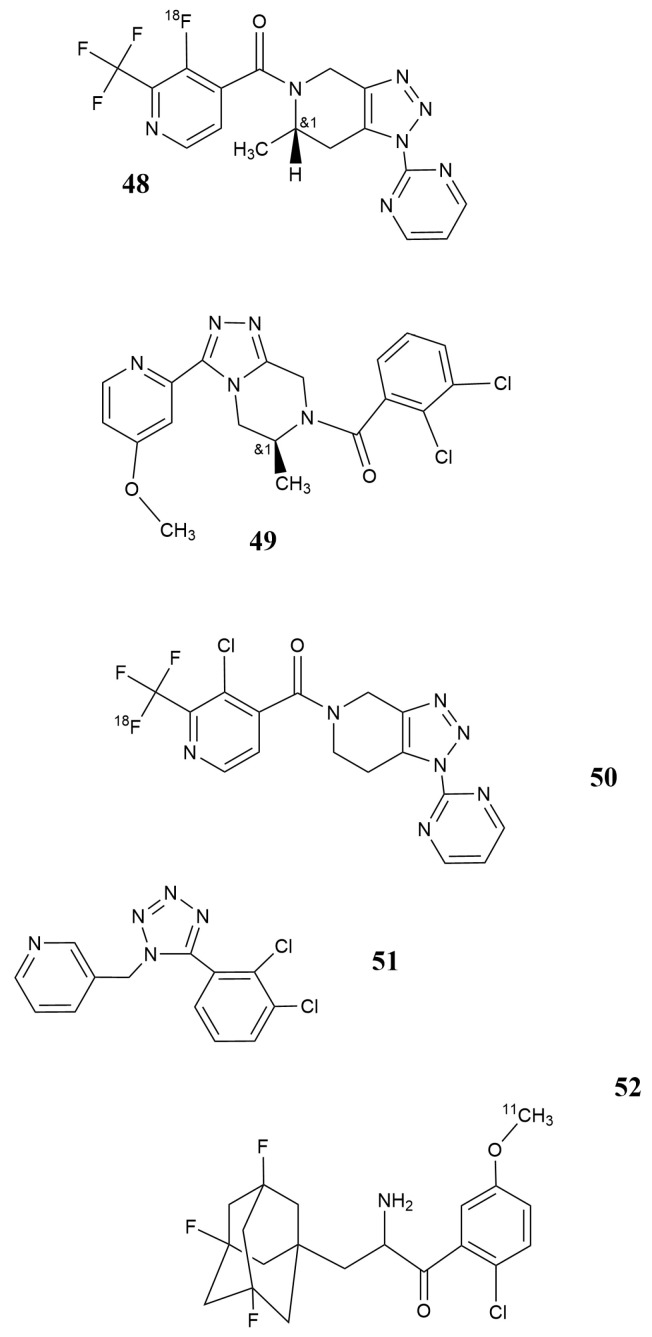
P2X7 receptor ligands **48**: [^18^F] JNJ-64413739; **49**: JNJ54173717; **50**: [^18^F]-PTTP; **51**: A438079; **52**: SMW139.

Meanwhile, a collection of further potential PET tracers occurred. The results of a phase 1 study were presented recently by Koole et al. [125] for the triazole derivative [^18^F] JNJ64413739 (Figure 11: [48]) developed at the Janssen laboratories [125,126,127,128]. The compound with appropriate log P of 2.05 and selectivity for the target receptor is regarded as a promising structure for further evaluation as a PET tracer.

A similar PET tracer (Figure 11: [49]) was reported for small animal investigations, PET in rhesus monkey [129] and, in 2019, for a first-in-human investigation in small groups of healthy volunteers and patients with Parkinson’s disease [130]. The report supplied data on dosimetry and biodistribution but could yet not confirm differences between healthy volunteers and patients. The authors presumed different genotypes in the small groups investigated as the cause of absent differences between ill and healthy persons.

The tetrazole P2X7 receptor antagonist A438079 (Figure 11: [51]) released already 2006 in the Abbott laboratories shares with A-740003 (Figure 10: [45]) some nociceptive abilities which have been described to be due to its anti-inflammatory properties, especially in chronic processes like arthritis [126,127,128]. The compounds were selected from a series of tetrazole derivatives [131,132,133], and cyanoguanidines [131] presented properties of competitive inhibition at the original binding site of endogenous ligands.

Finally, Janssen et al. [134] presented in 2018 out of a series of adamantanyl benzamides a C-11-labeled, high-affinity compound which interacts with the receptor as allosteric antagonists. The authors underline, however, the suitability of the ligand SMW139 (Figure 11: [52]) chosen for in vivo visualization.

### 2.8. TREM (Triggering Receptor Expressed on Myeloid Cells)

In contrast to the aforementioned receptors, for the triggering receptor expressed on myeloid cells 2 (TREM2) subtypes in the scope of small molecule interaction with brain cells, the knowledge is restricted to rather non-selective phospholipids [135].

There is, already, a lot known on TREM structures, on functional roles in the brain [136,137,138] and on a role as a potential site of mutations associated with Alzheimer’s disease, like Nasu Hakola disease or frontotemporal dementia. However, the access for PET probes to TREM2 as diagnostic tools in such diseases is still a relatively untouched field. TREM2 is a transmembrane protein with an extracellular v-type immunoglobulin domain and a short cytoplasmic domain [138,139,140,141,142]. It can act on spleen tyrosine kinase (SYK) mediated by interaction with the accessory adaptor DAP12 (DNAX activating protein of 12 kDa) and induces a signaling cascade involving the extracellular signal-regulated kinase (ERK1/2), phospholipase Cγ (PLCγ) and the E3 ubiquitin ligase scaffold protein, E3 ubiquitin ligase (Cbl) after phosphorylation steps in TREM2. Additionally, TREM is present also as a soluble version (soluble triggering receptor expressed on myeloid cells 2, sTREM2) which can be released by ADAM17, 10 and a gamma secretase [142]. TREM2 has been confirmed to provide binding place for LPS, phospholipids, high density lipoproteins (HDL) and low density lipoproteins (LDL) [36], and is expressed in microglia of the CNS, osteoclasts, alveolar and peritoneal macrophages as well as natural killer cells.

Mutations of TREM2 can have very different consequences for function and structure of the protein.

Homozygote mutations are known for a syndrome called Nasu Hakola disease—a disorder in the scope of leukodystrophie and lipodystrophies. It includes frontotemporal alterations of the brain with increasing dementia, degeneration of nerves and lipid tissue as well as a polycystic osteopathy [133].

Recent reports show a reduced activation of microglia in a model mice of Alzheimer’s disease combined with a knock-out of TREM2 [143]. Endogenous small molecule inhibitors of TREM2 are phosphatidylethanolamine and phosphatidylserine. Positron emitting tracers, however, have not been not introduced or subjected to clinical trials. Selective and appropriately sensitive small molecule ligands for brain PET of this structure are yet not available.

## 3. Discussion

Recently, the cerebellum was regarded as a preferable reference region of neuroradiological imaging [128,144,145]. However, new anatomical [26,146], cell biological and physiological approaches have moved it to a role in multiple functions for the organism and as an emancipated part of the brain.

Early radiological approaches with increasingly detailed analysis of inflammatory or degenerative processes of the cerebellum in more recent years included [^18^F] FDG [2,17,147,148,149] and ^15^O measurements or MRI methods [17,26,150].

Ganz et al. demonstrated [24] a heterogeneous expression of the different 5HT receptor subtypes and the transporter in cerebellar hemispheres, white matter and vermis in mice, and questioned the old role of the cerebellum as a reference region of bio-amine receptor imaging.

Additionally, conditions regarding the experimental side which could be of relevance for final results of neuroimaging studies are more and more objective of research in detail. These are, for instance, also the consequences of anesthetic treatments in the cerebrum and cerebellum. Bascunana et al. [149] provided a consistent evaluation of emission tomographic experimental models in mice and the role of some anesthetics commonly used in such approaches. The authors recognized not only differences in the general actions of anesthetics, but also between the influences on the cortex and the cerebellum regarding the uptake of FDG. For instance, mouse cerebellum showed after isoflurane and sevoflurane anesthesia a higher uptake, although isoflurane is known to reduce the cortical [^18^F] FDG uptake due to reduced phosphorylation of glucose. Ketamine and xylazine known to result in a lower uptake of FDG showed a higher cortical and lower cerebellar uptake. The authors compared the inverse correlation between blood glucose level and FDG uptake suggested already by Sokoloff in 1977 [151] and tested the action of insulin on metabolic rate and FDG uptake. They observed diverse regional differences in FDG uptake in the cortex reducing the total cortical uptake. Such information on approach-mediated alterations of the baseline in experimental models is not less important than the identification and visualization of new targets.

For almost all receptors in the focus of the present contribution, novel tracers have shown their uptake also in the cerebellum, in part, with a comparably high accumulation in cerebellar tissue, such as in the cerebrum. Examples are the CB1 receptor ligand [^18^F] MK9470, the CB2 receptor ligand [^18^F] NE40 or also ligands of NPY2 and P2Y12 receptors.

These observations, together with increasing sub-regional differentiation of cerebellar activation [24], provide a supportive background also for quantitative approaches to establish specific and sensitive methods of neuroimaging which allow also increasingly detailed investigation of the cerebellum.

Although with focus on the visualization of microglia as an indicator of inflammatory active processes, hitherto, the TSPO ligands continue to be the favored tool in PET imaging [92,152], it can be noted that there are some efforts in identification of targets and small molecule ligands which potentially differentiate between activated and resting microglia. Notably, P2Y12 receptor expression in microglia is a promising interesting target [28].

More potentially suitable PET tracers have been proposed and evaluated in initial animal experiments for the P2X7 receptor [113,126,127]. A small-molecule competitive antagonist was identified but has not yet been evaluated as a PET tracer [127]. Even if most other candidates have been developed for oral application, this competitive antagonist should improve the possibilities for exact quantification of the microglia and glia target receptor. Further allosteric and non-competitive candidate structures have been labeled with C-11 or F-18 and recommended for further testing. Suitability of allosteric small molecule compounds in diagnostic PET is also a question for visualization of several neuropeptide receptors.

However, the pipeline for ligands of purinoceptors is better filled, and there are more preclinical PET data than for the two immune receptors RAGE and TREM2 and its ligands.

The development of CX3CR1 ligands is particularly of interest because of potential therapeutic benefits expected from the recently proposed piperidin-1-yl compound JMS-17-2, shown to reduce the seeding and lodging of breast cancer cell in mice [81]. Moreover, an inhibitory action is reported in EAE models.

The CB2 receptor in the cerebellum is usually lower expressed than the CB1 receptor. However, it is known to be inducible in different disease entities like inflammation. Finally, predominantly, the methoxy-quinolinone derivative NE40 has been tested also in vivo in monkey and rat [72,73,74,77].

Generally, the broadest spectrum of new structures is provided in the field of sphingosine 1 phosphate receptor ligands. Here, also the diversity in side effects and on immune cells is well investigated. For therapeutics, possibly the careful selection of combinations in subtype selectivity can support the access to successful treatment of demyelination diseases. For PET and in regard to pharmacokinetic properties, some promising candidates have been identified, such as W146, VPC-44116, VPC 23015 or CS-0777.

Finally, all tracers applied in animal experiments and accumulated in the brain could achieve also the cerebellum. They provide today already valuable additional tools for investigation and understanding of the role of this region of the brain and related diseases.

## 4. Conclusions

Although few quantitative data on receptor protein content in cerebellar tissue or native microglia are available, some of the PET investigations of the microglial receptors confirm the presence also in cerebellar tissue regions.

This is underlined by experimental PET data reporting on comparable binding of tracers for CB2, NPY2 and P2X7 receptors in the cerebral cortex and the cerebellum, even if this is rather a semi-quantitative approach.

One of the most interesting challenges is the question for candidate targets and respective receptor ligands allowing for the discrimination between pro- and anti-inflammatory functional types of microglial cells, especially as an advantage in competition with TSPO ligands presently employed for PET in inflammatory tissues. Experimentally, P2Y12 receptor overexpression was shown in activated M2-type (anti-inflammatory) microglia [28]. One potential small molecule PET tracer has been proposed with [^11^C] C5 and might be a first lead structure for proof of concept, although the structure requires pharmacokinetic optimization.

The challenge for pharmacokinetic optimization is also valuable for the proposed ligands of the immune receptor RAGE and the chemokine receptor CX3XR. The latter can be regarded instantly as the candidate structure among the potential marker receptors of microglia with the highest specificity.

The optimization of the ligands for immune receptors, the CX3CR1 receptor and the P2X7 receptor can be also supported by molecular dynamics studies. These could supply essential knowledge also on the role of polymorphisms in pathophysiology, binding mode and efficiency of potential radiotracers for the receptor.

The competitive P2X7 receptor ligand (Figure 11 [51]) provides a promising lead structure for potential radiotracers. The use of the allosteric tracers, already available as radiolabeled compounds, poses the question of usability of such structures not only as potential therapeutics but also as PET diagnostic tools. In the discussion on neuronal expression versus the microglial dominating receptor, current data support specificity for glia cells, and in the brain, predominantly for microglia. [118,119].

Sphingosine 1 phosphate receptors are abundant in the brain but generally less specific for microglia than other receptors. Selective P2X7 receptor ligands like selective NPY2 and CB2 receptor ligands show in the cerebellum a similar uptake to that in the cerebral cortex, even if differences of in vivo expression cannot yet not confirmed clinically for pathological conditions. The endogenous in vivo expression of the purinoceptor P2X7 postulated for neurons could not be confirmed convincingly [117,118,119] to date, but the expression has been ensured in Bergman glia [152,153]—a specialized type of astrocytes of the cerebellum, which envelop Purkinje neurons.

## Abbreviations

**ASD**	Autism spectrum diseases
**Bbb**	Blood–brain barrier
**Cbl**	Scaffold protein, E3 ubiquitin ligase
**CRISPR**	Clustered regularly interspaced short palindromic repeats
**CX3CR1**	Chemokine receptor CX3C; fractalkine receptor
**DAP12**	DNAX activating protein of 12 kDa
**EAE**	Experimental autoimmune encephalitis
**eNOS**	Endothelial NO synthase
**FDG**	Fluorodeoxyglucose
**GIRK**	G-protein coupled inwardly rectifying potassium channel
**GPCR**	G-protein-coupled receptor
**HDAC**	Histone deacetylase
**htert Human**	telomerase reverse transcriptase
**LRR**	Leucine-rich repeat domain
**MerTK**	Myeloid-epithelial reproductive tyrosine kinase
**NLRP3**	NOD-, LRR- and pyrin domain-containing protein 3
**NOD**	Nucleotide binding oligomerization domain-containing protein 2
**PET**	Positron emission tomography
**RAGE**	Receptor for advanced glycation end products
**S1P**	Sphingosine 1phosphate
**sTREM2**	Soluble triggering receptor expressed on myeloid cells 2
**SUV**	Standard uptake value
**TREM2**	Triggering receptor expressed on myeloid cells 2
**TRM**	Tissue resident memory cells
**TSPO**	18 kDa-translocator protein

## Appendix A

Legends to Figure 2, Figure 3, Figure 4, Figure 5, Figure 6, Figure 7, Figure 8, Figure 9, Figure 10 and Figure 11 (IUPAC names obtained using ChemDraw 19.0).

Figure 2. Sphingosine 1 phosphate receptor ligands.

1. W146: *rac*-(*R*)-(3-amino-4-((3-hexylphenyl)amino)-4-oxobutyl)phosphonic acid; CAS RN^®^ 909725-61-7
2. VPC44116: *rac*-(*R*)-(3-amino-4-((3-octylphenyl)amino)-4-oxobutyl)phosphonic acid; CAS RN^®^ 1161429-70-4
3. VPC23019: *rac*-(*R*)-2-amino-3-((3-octylphenyl)amino)-3-oxopropyl dihydrogen phosphate; CAS RN^®^ 449173-19-7
4. VPC22173: *rac*-(*R*)-2-amino-3-((4-octylphenyl)amino)-3-oxopropyl dihydrogen phosphate; CAS RN^®^ 449173-10-8
5. VPC22277: *rac*-(*R*)-2-amino-3-((4-nonylphenyl)amino)propyl dihydrogen phosphate; CASRN^®^ 449173-15-3
6. VPC 3090: (1-amino-3-(3-octylphenyl)cyclobutyl)methanol;
7. VPC3090P: (1-amino-3-(3-octylphenyl)cyclobutyl)methanol phosphate;
8. CS0777: *rac*-(*R*)-1-(5-(3-amino-4-hydroxy-3-methylbutyl)-1-methyl-1H-pyrrole-2-yl)-4-(l-alanine.)butan-1-one; CAS RN^®^ 827344-05-8
9. CS0777P: *rac*-(*R*)-2-amino-2-methyl-4-(1-methyl-5-(4-(*p*-tolyl)butanoyl)-1H-pyrrole-2-yl)butyl dihydrogen phosphate; CAS RN^®^ 840523-39-9

Figure 3. Sphingosine 1 phosphate receptor ligands.

10. GSK2018682: 4-(4-(5-(5-chloro-6-isopropoxypyridin-3-yl)-1,2,4-oxadiazol-3-yl)-1H-indol-1-yl)butanoic acid; CAS RN^®^ 1034688-30-6
11. TASPO277308: (*R*)-3,4-dichloro-*N*-(1-(4-ethyl-5-(3-(4-methylpiperazin-1-yl)phenoxy)-4H-1,2,4-triazol-3-yl)ethyl)benzenesulfonamide; CAS RN^®^ 945725-50-8
12. TZ3321: 3-((2-fluoro-4-(5-(2′-methyl-2-(trifluoromethyl)-[1,1′-biphenyl]-4-yl)-1,2,4-oxadiazol-3-yl)benzyl)amino)-2-(methyl-11C)propanoic acid;
13. XLS541: *rac*-(*R*)-1-(2-(1H-1,2,3-triazol-1-yl)acetyl)-4-(4-fluorobenzyl)-*N*-(4-(4-fluorophenoxy)phenyl)-2,3-dihydro-1H-pyrrole-2-carboxamide;
14. Ex26: 1-(5′-((1-(4-chloro-3-methylphenyl)ethyl)amino)-2′-fluoro-3,5-dimethyl-[1,1′-biphenyl]-4-carboxamido)cyclopropane-1-carboxylic acid;
15. AUY954: 3-(((2-(2-(trifluoromethyl)-[1,1′-biphenyl]-4-yl)benzo[b]thiophen-5-yl) methyl)amino)propanoic acid; CAS RN^®^ 820240-77-5
16. Cl2: sodium 2-(4-ethoxyphenoxy)-5-(3-nonadecylcyclopenta-1,3-dien-1-yl)benzenesulfinate;
17. Prodrug 14: *rac*-methyl (*R*)-2-(3′-((4-chloro-2,5-dimethylphenyl)sulfonamido)-*N*,3,5-trimethyl-[1,1′-biphenyl]-4-carboxamido)-4-(dimethylamino)butanoate;
18. NIBR0213: *rac*-(3′-(((*R*)-1-(4-chloro-3-methylphenyl)ethyl)amino)-3,5-dimethyl-[1,1′-biphenyl]-4-carbonyl)-l-alanine.

Figure 4. Sphingosine 1 phosphate 2 receptor antagonists.

19. JTE013: *N*-(2,6-dichloropyridin-4-yl)-2-(4-isopropyl-1,3-dimethyl-1H-pyrazolo[3,4-b]pyridin-6-yl)hydrazine-1-carboxamide; CAS RN^®^ 383150-41-2
20. AB1: 2-(1-allyl-4-isopropyl-3-methyl-1H-pyrazolo[3,4-b]pyridin-6-yl)-*N*-(2,6-dichloropyridin-4-yl)hydrazine-1-carboxamide.

Figure 5. Cannabinoid receptor ligands.

CB1:

21. SR1441716 (Rimonabant): 5-(4-Chlorophenyl)-1-(2,4-dichlorophenyl)-4-methyl-*N*-1-piperidinyl-1H-pyrazole-3-carboxamide;
22. JHU-75528: 4-Cyano-1-(2,4-dichlorophenyl)-5-(4-methoxyphenyl)-*N*-1-piperidinyl-1H-pyrazole-3-carboxamide; CAS RN^®^ 947696-17-5

CB2:

23. NE 40: 8-butoxy-3-(2-cyclohexylacetyl)-7-methoxyquinolin-2(1H)-one;
24. SR144528: 5-(4-chloro-3-methylphenyl)-1-[(4-methylphenyl)methyl]-*N*-[(1*S*,2*S*,4*R*)-1,3,3-trimethylbicyclo[2.2.1]hept-2-yl]-1H-Pyrazole-3-carboxamide; CAS RN^®^ 192703-06-3

Figure 6. P2Y12 antagonists.

25. AZD-6140: (Ticagrelor), 3-[7-[[(1*R*,2*S*)-2-(3,4-difluorophenyl)cyclopropyl]amino]-5-(propylthio)-3H-1,2,3-triazolo [4,5-d]pyrimidin-3-yl]-5-(2-hydroxyethoxy)-, (1*S*,2*S*,3*R*,5*S*)-1,2-Cyclopentanediol; CAS RN^®^ 274693-27-5
26. C2: Ethyl 4-(3-(3-((5-chlorothiophen-2-yl)sulfonyl)ureido)azetidin-1-yl)-3-cyanobenzoate;
27. C5: Ethyl 6-(3-(3-((5-chlorothiophen-2-yl)sulfonyl)ureido)azetidin-1-yl)-5-cyano-2-methylnicotinate.

Figure 7. Antagonists at CX3CR1 and allosteric modulators.

28. AZD8797: 2-((2-amino-5-((1-phenylethyl)thio)thiazolo[4,5-d]pyrimidin-7-yl)amino)-4-methylpentan-1-ol; CAS RN^®^ 1586818-99-6
29. FBTTP: (*R*)-2-((2-amino-5-((2-fluorobenzyl)thio)thiazolo[4,5-d]pyrimidin-7-yl)amino)-4-methylpentan-1-ol;
30. JMS-17-2: 5-(3-(4-(4-chlorophenyl)piperidin-1-yl)propyl)pyrrolo[1,2-a]quinoxalin-4(5H)-one;
31. JMS_17modified: 5-(3-(4-(4-fluorophenyl)piperidin-1-yl)propyl)-4,5-dihydro-[1,2,3]triazolo[1,5-a]quinoxalin-4-ol.

Figure 8. NPY2 receptor ligands.

32. *N*-^11^C-Methyl-JNJ-31020028: *N*-(4-(4-(2-(diethylamino)-2-oxo-1-phenylethyl)piperazin-1-yl)-3-fluorophenyl)-*N*-(methyl-11C)-2-(pyridin-3-yl)benzamide; CAS RN^®^ 1094873-14-9
33. SF-11: *N*-(4-ethoxyphenyl)-4-phenylpiperidine-1-carbothioamide;
34. SF 31: 2-(2-methoxyphenyl)-1-((4-(5-(3-methoxyphenyl)-1,2,4-oxadiazol-3-yl) phenyl)amino)ethan-1-ol.

Figure 9. RAGE ligands.

35. [^18^F]RAGER: *N*-benzyl-*N*-cyclohexyl-4-(fluoro-^18^F)benzamide;
36. FPS-ZM1: *N*-benzyl-4-chloro-*N*-cyclohexylbenzamide;
37. Azeliragon: 3-(4-(2-butyl-1-(4-(4-chlorophenoxy)phenyl)-1H-imidazol-4-yl)phenoxy)-*N*,*N*-diethylpropan-1-amine; CAS RN^®^ 603148-36-3
38. cmpd2: 5-butyl-4-(4-(4-chlorophenoxy)phenyl)-2-(4-(2-(4-methylpiperazin-1-yl)ethoxy)phenyl)thiazole;
39. cmpd 3: 4,6-bis(4-chlorophenyl)-*N*-(3-(2-(diethylamino)ethoxy)phenyl)pyrimidin-2-amine;
40. cmpd 4: 4,6-bis(4-chlorophenyl)-*N*-(2-(2-(diethylamino)ethoxy)phenyl)pyrimidin-2-amine;
41. cmpd 5: 4,6-bis(4-chlorophenyl)-*N*-(3-(2-(3-(dimethylamino)pyrrolidin-1-yl)ethoxy)phenyl)pyrimidin-2-amine;
42. cmpd 6: *N*-(2-((3-ethylpentyl)oxy)-5-methoxyphenyl)-4,6-bis(4-fluorophenyl)pyrimidine-2-carboxamide.

Figure 10. P2X7 receptor ligands.

43. [^18^F]EFB: (*E*)-2-cyano-1-(4-(fluoro-^18^F)benzyl)-3-(quinolin-5-yl)guanidine;
44. GSK-1482160: *N*-[[2-chloro-3-(trifluoromethyl)phenyl]methyl]-1-methyl-5-oxo-2-Pyrrolidine carboxamide; CAS RN^®^ 1001389-72-5
45. A-740003: *N*-[1-[[(Cyanoamino)(5-quinolinylimino)methyl]amino]-2,2-dimethylpropyl]-3,4-dimethoxybenzeneacetamide; CAS RN^®^ 861393-28-4
46. KN-04: 4-[2-[(5-isoquinolinylsulfonyl)amino]-3-(4-phenyl-1-piperazinyl)propyl]phenylester-5-Isoquinolinesulfonic acid; CAS RN^®^ 129695-80-3
47. KN-62: 5-Isoquinolinesulfonic acid, 4-[(2*S*)-2-[(5-isoquinolinylsulfonyl) methyl amino]-3-oxo-3-(4-phenyl-1-piperazinyl)propyl]phenyl ester; CAS RN^®^ 127191-97-3

Figure 11, P2X7 receptor ligands part 2.

48. [^18^F]JNJ64413739: *rac*-(*S*)-(3-(fluoro-^18^F)-2-(trifluoromethyl)pyridin-4-yl)(6-methyl-1-(pyrimidin-2-yl)-1,4,6,7-tetrahydro-5H-[1,2,3]triazolo[4,5-c]pyridin-5-yl)methanone;
49. JNJ54173717: *rac*-(*R*)-(2,3-dichlorophenyl)(3-(4-methoxypyridin-2-yl)-6-methyl-5,6-dihydro-[1,2,4]triazolo[4,3-a]pyrazin-7(8H)-yl)methanone;
50. [^18^F]-PTTP: (3-chloro-2-(difluoro(fluoro-^18^F)methyl)pyridin-4-yl)(1-(pyrimidin-2-yl)-1,4,6,7-tetrahydro-5H-[1,2,3]triazolo[4,5-c]pyridin-5-yl)methanone;
51. A438079: 3-((5-dichlorophenyl)-1H-tetrazol-1-yl)methyl)pyridine; CAS RN^®^ 899431-18-6
52. [^11^C]SMW139: 2-amino-1-(2-chloro-5-(methoxy-^11^C)phenyl)-3-((3s,5s,7s)-3,5,7-trifluoro-adamantan-1-yl) propan-1-one.
